# COVID-19 Vaccination and Public Health Countermeasures on Variants of Concern in Canada: Evidence From a Spatial Hierarchical Cluster Analysis

**DOI:** 10.2196/31968

**Published:** 2022-05-31

**Authors:** Daniel A Adeyinka, Cory Neudorf, Cheryl A Camillo, Wendie N Marks, Nazeem Muhajarine

**Affiliations:** 1 Department of Community Health and Epidemiology College of Medicine University of Saskatchewan Saskatoon, SK Canada; 2 Saskatchewan Population Health and Evaluation Research Unit Saskatoon, SK Canada; 3 Johnson Shoyama Graduate School of Public Policy University of Regina Regina, SK Canada

**Keywords:** COVID-19, variants of concern, stringency index, mobility index, vaccination coverage, machine learning, Canada

## Abstract

**Background:**

There is mounting evidence that the third wave of COVID-19 incidence is declining, yet variants of concern (VOCs) continue to present public health challenges in Canada. The emergence of VOCs has sparked debate on how to effectively control their impacts on the Canadian population.

**Objective:**

Provincial and territorial governments have implemented a wide range of policy measures to protect residents against community transmission of COVID-19, but research examining the specific impact of policy countermeasures on the VOCs in Canada is needed. Our study objective was to identify provinces with disproportionate prevalence of VOCs relative to COVID-19 mitigation efforts in provinces and territories in Canada.

**Methods:**

We analyzed publicly available provincial- and territorial-level data on the prevalence of VOCs in relation to mitigating factors, summarized in 3 measures: (1) strength of public health countermeasures (stringency index), (2) the extent to which people moved about outside their homes (mobility index), and (3) the proportion of the provincial or territorial population that was fully vaccinated (vaccine uptake). Using spatial agglomerative hierarchical cluster analysis (unsupervised machine learning), provinces and territories were grouped into clusters by stringency index, mobility index, and full vaccine uptake. The Kruskal-Wallis test was used to compare the prevalence of VOCs (Alpha, or B.1.1.7; Beta, or B.1.351; Gamma, or P.1; and Delta, or B.1.617.2 variants) across the clusters.

**Results:**

We identified 3 clusters of vaccine uptake and countermeasures. Cluster 1 consisted of the 3 Canadian territories and was characterized by a higher degree of vaccine deployment and fewer countermeasures. Cluster 2 (located in Central Canada and the Atlantic region) was typified by lower levels of vaccine deployment and moderate countermeasures. The third cluster, which consisted of provinces in the Pacific region, Central Canada, and the Prairies, exhibited moderate vaccine deployment but stronger countermeasures. The overall and variant-specific prevalences were significantly different across the clusters.

**Conclusions:**

This “up to the point” analysis found that implementation of COVID-19 public health measures, including the mass vaccination of populations, is key to controlling VOC prevalence rates in Canada. As of June 15, 2021, the third wave of COVID-19 in Canada is declining, and those provinces and territories that had implemented more comprehensive public health measures showed lower VOC prevalence. Public health authorities and governments need to continue to communicate the importance of sociobehavioural preventive measures, even as populations in Canada continue to receive their primary and booster doses of vaccines.

## Introduction

### Background

The devastating impacts of COVID-19 cannot be overemphasized. With an estimated 423 million cases and 6 million deaths (as of February 21, 2022) worldwide [1], the pandemic is one of the worst in human history. In Canada alone, about 3.2 million people, representing 8.4% of residents, have been infected with COVID-19 [2]. Canada’s case fatality rate for COVID-19 is 1.1% (ie, 36,000 deaths) [2].

At the time our analyses were performed (June 15, 2021, when the third wave was waning), there were 3 forces in “tension”: vaccine uptake, newly emerging variants of COVID-19, and calls to “re-open the economy.” Since then, the emergence and spread of additional variants of concern (VOCs) and the expansion of vaccination campaigns have changed the complexion of the pandemic. Variants, as expected, have added complexity to the nature of COVID-19. At the same time, following a slow start to vaccination rollouts, vaccine uptake has been increasing in Canada, and many provinces and territories, who are largely responsible for vaccination and public health policy in Canada’s decentralized federation, have become eager to relax public health countermeasures. However, public health experts remain uneasy about variant-led surges and outbreaks and are calling for the reapplication of some measures.

As of February 21, 2022, 5 phylogenetic VOCs declared by the World Health Organization are being tracked in Canada. The Alpha (B.1.1.7), Beta (B.1.351), Gamma (P.1), Delta (B.1.617.2), and Omicron (B.1.1.529) variants, first detected in the United Kingdom, South Africa, Brazil, India, and South Africa, respectively, are spreading in Canada and the rest of the world [3]. Canada confirmed its first cases of the Alpha variant in a couple from Toronto who had contact with a traveler from the United Kingdom on December 26, 2020 [3]. The Beta variant was first reported in Alberta on January 8, 2021 [3]. The Gamma variant was first confirmed in Ontario in an international traveler from Brazil on February 8, 2021 [3]. On April 4, 2021, the Delta variant was first reported in British Columbia [3]. The latest variant (Omicron) was first detected in Ontario from 2 travelers from Nigeria on November 28, 2021 [3].

Although much still remains to be learned about the epidemiology, diagnosis, management, and sequalae of these 5 VOCs, using the data available at the time of analysis, we sought to understand the spread of VOCs in Canada and how they might be held in check by vaccine uptake and public health countermeasures. Although lab-based research and modeling have shown the need to combine public health countermeasures with vaccination to achieve epidemic control [4,5], there has been a paucity of population-level studies to assess the effects of nonpharmaceutical public health measures on VOCs. In a mathematical model of COVID-19 transmission in New York City, public countermeasures such as mask wearing and social distancing were shown to have immediate impact on the epidemic [5]. The synergistic effects of vaccination and public countermeasures in reducing new COVID-19 cases cannot be overemphasized [5]. Alpha and Delta variants are more transmissible than the initial strains of the virus, but the Gamma, Beta, and Delta variants hugely impact vaccine effectiveness. A major concern is the continuous genetic evolution of the virus, which complicates reopening plans across Canada. Although studies are underway to determine the degree of virulence of Omicron variants, it is believed that a subvariant known as BA.2 is more contagious than its predecessor, BA.1, and currently dominating in Canada [6].

### Objectives

This “up to the point” analysis of VOCs in Canada (ie, during the downward trajectory of the third wave) examines (1) clustering patterns of COVID-19 mitigation efforts and (2) cluster differences in the prevalence of COVID-19 VOCs in Canada. In doing so, it aimed to provide insights into the differences in the subnational responses to inform ongoing policy and public health interventions at the provincial and territorial levels of government.

## Methods

### Ethics Approval

This analysis centered on publicly available data with no identifiable information about the people studied. Therefore, research ethics board approval was not required for this study.

### Data Sources

We analyzed provincial- and territorial-level data on COVID-19 VOCs in Canada along with data on COVID-19 mitigating strategies from publicly available data sources. Our outcome variable—prevalence of VOCs by type and total—was estimated as the proportion of cases with VOC per 1 million population as of June 15, 2021. The cumulative number of VOC cases for Alpha, Beta, Gamma, and Delta variants was extracted from a COVID-19 VOC tracker in Canada [7]. Populations at risk were predefined as the first quarter, 2021, provincial population estimates obtained from Statistics Canada [8]. Our independent variables—vaccine uptake (the percentage of each provincial or territorial population fully vaccinated against COVID-19), policy response (stringency index—see the following paragraphs for more details), and behavioral changes (mobility index) were mapped with the VOC prevalence outcome. These mitigating factors were selected based on growing evidence that uptake of primary series of vaccination (2 doses) [9-15], reduction in human mobility [16-18], and social distancing policies [19,20] are effective in curtailing community transmission of COVID-19.

During the study period, 3 COVID-19 vaccines (ie, AstraZeneca, Moderna, and Pfizer-BioNTech) were authorized and in use in vaccination campaigns in Canada. Full vaccination coverage rates were retrieved from the COVID-19 Tracker Canada [21]. As opposed to first dose (ie, partial immunization) rates, full vaccination (2 doses) rates were analyzed in this study because complete vaccination is widely believed to offer greater protection than partial vaccination in slowing down COVID-19 transmission, hospitalizations, disease sequalae, and fatalities [22].

The stringency index is a composite score generated by the researchers at the University of Oxford to document how the governments’ coronavirus responses are changing around the world and over time. The metric is an additive score of 9 indicators (ie, school and workplace closures, restrictions on public transport, cancellation of public events and gatherings, stay-at-home policies, travel restrictions, public information campaigns, testing policies, contact tracing, and masking), measured on an ordinal scale, and rescaled to vary from 0 to 100. The lowest possible score is 0 (mildest), and the highest score is 100 (strictest). The policy index was publicly available at the Oxford COVID-19 Government Response Tracker (OxCGRT) website [23]. The constituent variables used in generating the stringency index are summarized in Multimedia Appendix 1. It should be noted that the stringency index is not a measure of effectiveness of government policies in response to COVID-19 but rather a measure of the degree to which and the comprehensiveness of governmental response. To account for the changing patterns of COVID-19 containment policies, average stringency indices for the period of January 1, 2021, to June 15, 2021 were reported.

To assess individuals’ compliance with government stringency measures (especially reduction in human movement to slow the spread of the virus), we obtained mobility reports from Google LLC [24]. Recent infodemiological and infoveillance studies [25-29] have utilized mobility reports from Google [24] to observe changes in the movement of people to places designed as high risk based on relative frequency, time, and duration of visits during the pandemic. The technical details of data aggregation and anonymization procedures have been fully described by Aktay et al [30]. Through Google Map’s location history feature, daily anonymized data on people’s movement to places such as retail and recreation, grocery stores and pharmacies, parks, transit stations, workplaces, and residential areas were collected from smartphones and compared to the baseline 5-week prepandemic period (January 3, 2020, to February 6, 2020). Due to privacy issues, Google could not provide information on the inter- or intraprovincial and territorial movement [30]. Also, in a bid to ensure additional privacy protections, a metric for a given place is discarded when the counts of the opted-in Google users are less than 100 people or the geographical area is less than 30 km^2^ [30]. As deemed useful by public health researchers to make critical decisions about COVID-19, the Google community mobility reports help provide insights into how busy certain places are and, thus, the extent to which individuals are engaging in social distancing [30].

To determine mobility patterns across the provinces and territories, we estimated the average number of visits to each category of place (eg, park or workplace) between January 1, 2021, and June 15, 2021. The average of the mobility patterns across the entire study period was estimated to account for changes in movement due to weather and holidays, as well as any within-province variations of public health countermeasures. Using the first component of principal component analysis (PCA), the dimensions of the 6 variables that measured changes in movement of people relative to the pre-COVID era were reduced with the singular value decomposition method and z-score transformation (ie, mean of 0 and variance of 1). The first component (interpreted as the mobility index) explained 60% of the total variance, and its eigenvalue was 3.60. The first component had positive loadings on residential areas (0.47) and parks (0.36), but negative loadings on workplace (–0.5), transit stations (–0.49), grocery stores (–0.11), and retail or recreational centers (–0.39). For each variable, a positive loading suggests a higher mobility index, and negative loading indicates a lower mobility index.

### Statistical Analysis

Descriptive statistics were generated. We conducted a spatial agglomerative hierarchical cluster analysis (unsupervised machine learning) to detect clusters of spatial (dis)similarities in COVID-19 mitigating factors in GeoDa version 1.18 software [31]. Furthermore, we determined differences in prevalence of VOCs across the clusters. A symmetrical distance-based weight matrix with an optimal arc distance of 7000 km was generated. After many calibrations, single-linkage clustering with an Euclidean distance function and geometric centroid weight of 1 was considered appropriate and used.

A spatial hierarchical cluster analysis was performed using the mitigating factors (ie, vaccine uptake, public health countermeasures, and mobility). With the single linkage, intercluster distance was determined by the closest distance between the observations (ie, closest neighbor clustering). The first step was to transform the independent variables using z-score standardization since they were in different scales. Z-score standardization is an important preprocessing step for a machine learning algorithm, which involves rescaling the features to have a normal distribution. Data standardization before PCA has been shown to outperform an unscaled data set [32]. In an attempt to select the number of clusters that provided the best fit (distinct clustering), we used a stopping rule—the Duda-Hart index. We selected groups with the highest Duda-Hart index (0.6) and lowest pseudo T-squared (4.4). The stopping rule corresponds to the 3 clusters reported. 

**Figure 1:**
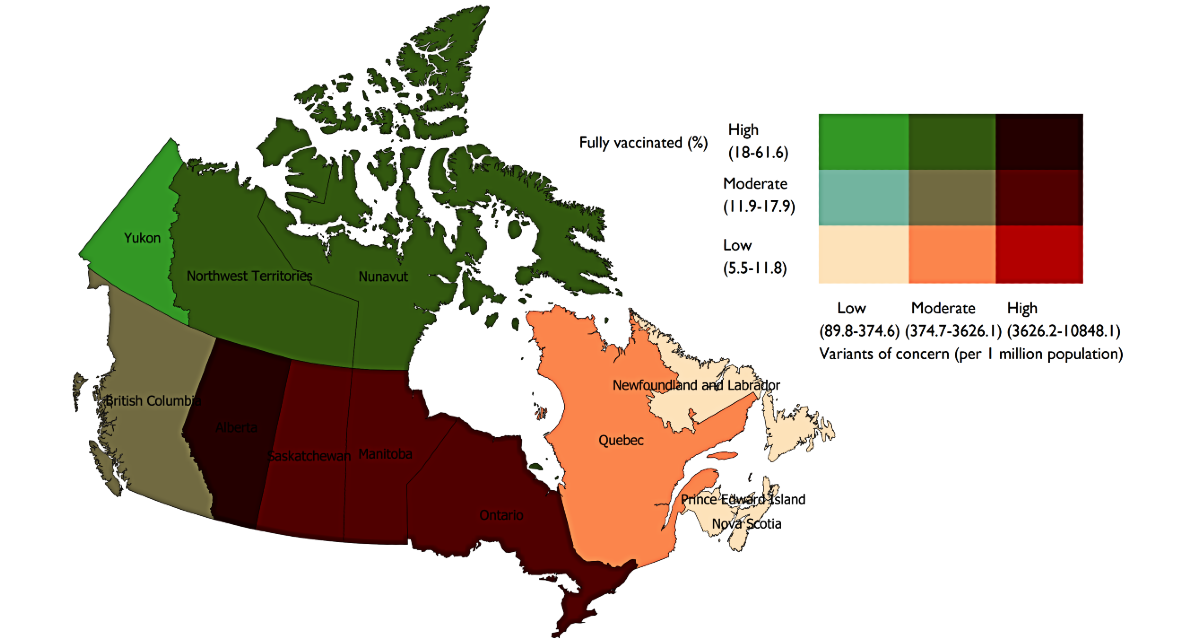
Association between variants of concern and 2 doses of COVID-19 vaccine in Canada as of June 15, 2021.

**Figure 2:**
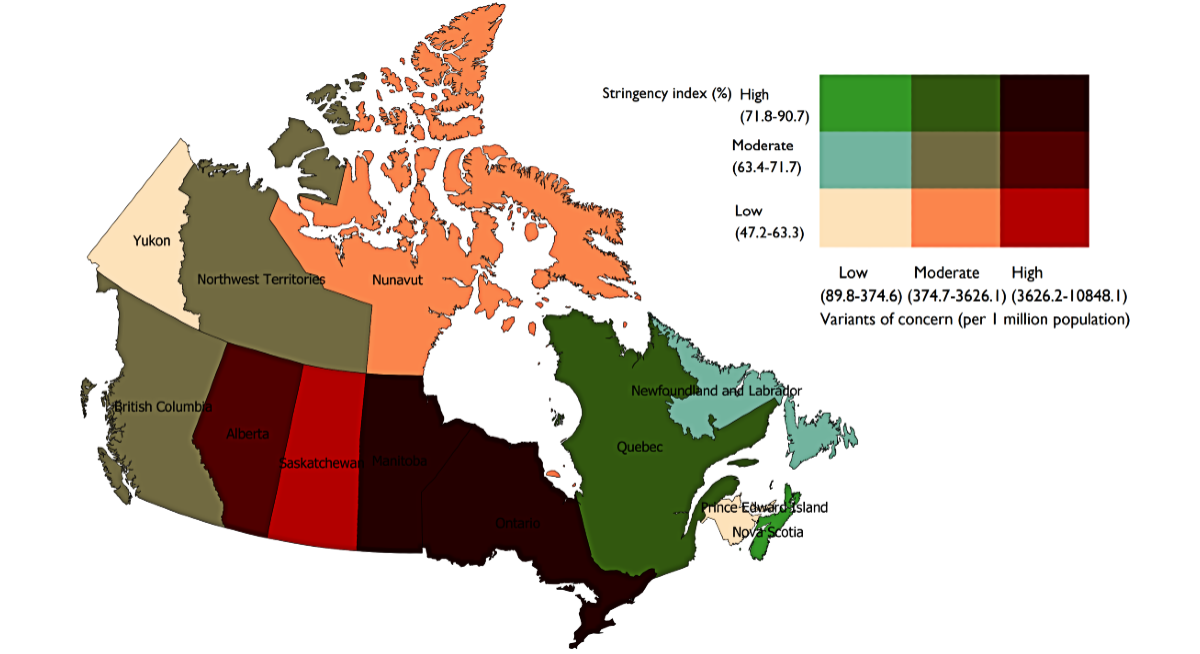
Association between variants of concerns and stringency measures in Canada as of June 15, 2021.

**Figure 3:**
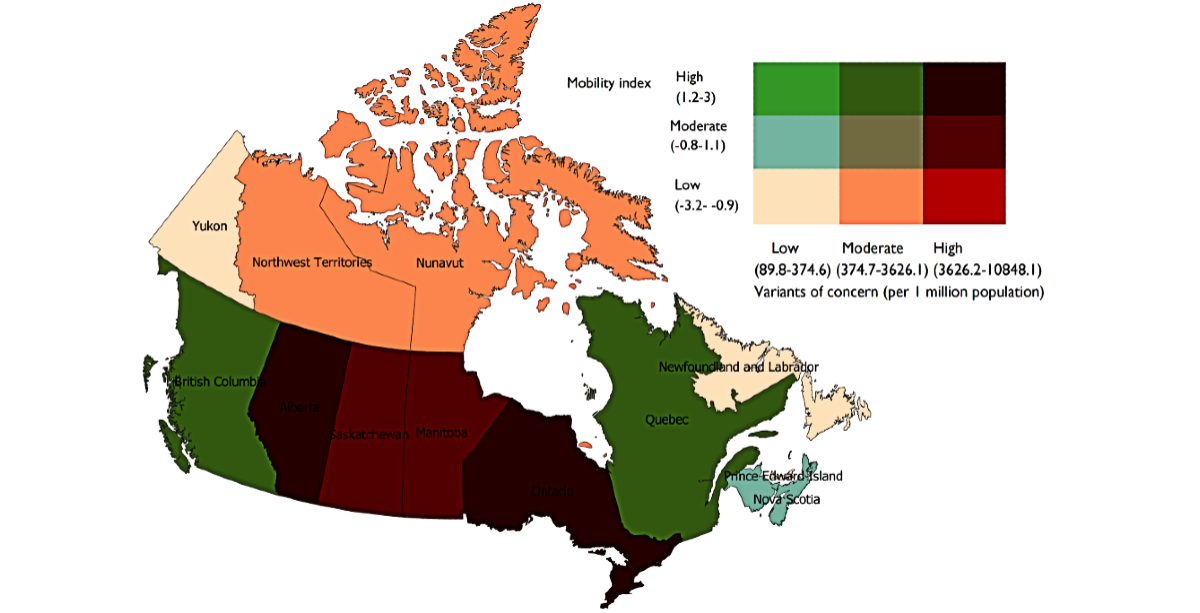
Association between variants of concern and mobility index in Canada as of June 15, 2021.

The Kruskal-Wallis equality-of-population rank test was used to determine the differences in the prevalence of VOCs among the clusters using Stata version 17.0 software [33]. A rank-based nonparametric test was used because the sample size is small (ie, 11 provinces and territories). Post hoc pairwise (posteriori) comparisons of the clusters were performed with the Dunn test; false detection rates were minimized by using Benjamini-Hochberg adjustment. The statistical significance was set at 2-sided *P*<.05. To visualize the relationships between the prevalence of VOCs, vaccine uptake, and countermeasures, bivariate choropleth maps were generated in QGIS version 3.12.1 software [34]. The bivariate maps were based on a quantile classification (ie, tertile); see Figures 1-3. As shown, the 3x3 2D color palette density becomes progressively darker as it moves from lower to higher tertiles and highlights the differences in relative position of features. The tertile classification is based on the sample distribution. We report the observed value ranges for each tertile classification.

## Results

### Descriptive Statistics

Table 1 shows the distribution of COVID-19 VOCs in Canada. As of June 15, 2021, when our analysis was performed, 4 VOCs (Alpha, B.1.1.7; Beta, B.1.351; Gamma, P.1; and Delta, B.1.617.2) had been identified, for a prevalence of 6157.5 per 1 million population and 16.7% of all cases. Nova Scotia reported the lowest, at 89.9 per 1 million population, to Alberta the highest, at 10,848.1 per 1 million population. At 91.4% of all VOCs, the Alpha variant was the predominant strain in Canada. The Gamma variant accounted for 6.5%, Beta variant for 0.8%, and Delta variant for 0.8% of the mutant strains. Although Alberta and Ontario had a higher prevalence of the Alpha variant, lower prevalence of this strain was observed in Yukon and Nova Scotia. The prevalence of the Beta variant was highest in Ontario, followed by Quebec. The Gamma and Delta variants were more common in British Columbia and Alberta.

Compared with the baseline (January 3, 2020-February 6, 2020), mobility related to home or residential areas increased by 12.7% between January 1, 2021, and June 15, 2021, among Canadians. On average, movement of people to parks and outdoor spaces increased by 38.8%; however, in Prince Edward Island, it decreased (average trend=–45%). Overall, mobility related to visits to grocery or pharmacy stores decreased by 4.6% across Canada but increased in Nova Scotia (by 2.6%), British Columbia (2.5%), and Saskatchewan (1.9%). Across Canada, movement related to public transport stations decreased by 56.5%, retail and recreational centers by 29.4%, and workplaces by 31.3%. The average national stringency index for COVID-19 was 73.6% (lowest in Yukon at 47.2% and highest in Ontario at 90.7%). The Canadian population fully vaccinated against COVID-19 was 13.8% (lowest in Newfoundland and Labrador at 5.7% and highest in Yukon at 61.6%).

**Table 1:** Geographic-specific distribution of COVID-19 variants of concern in Canada, June 15, 2021 (per 1,000,000 population).

Location	Overall	Alpha B.1.1.7	Beta B.1.351	Gamma P.1	Delta B.1.617.2
Nunavut	532.9	532.9	0	0	0
Newfoundland and Labrador	374.68	359.31	11.53	1.92	1.92
Prince Edward Island	175.2	162.68	0	0	12.51
Nova Scotia	89.85	74.53	12.25	1.02	2.04
New Brunswick	236.55	230.16	5.11	1.28	0
Quebec	900.43	791.63	47.81	56.9	4.08
Ontario	9919.34	9530.06	77.06	280.38	31.85
Manitoba	4597.61	4371.68	32.59	120.21	73.14
Saskatchewan	5461.34	5200.06	8.48	195.96	56.84
Alberta	10,848.11	10,122.95	35.16	609.52	80.47
British Columbia	3626.21	1963.5	26.2	1465.35	171.16
Yukon	189.61	71.10	0	118.51	0
Northwest Territories	1705.96	1683.8	0	22.16	0
Canada (overall)	6157.47	5655.01	50.33	401.75	50.38

### Spatial Hierarchical Clustering

Cluster analysis with the single-linkage method identified 3 cluster profiles of VOC prevalence, vaccine uptake, public health countermeasures, and mobility among Canadian provinces or territories (see Table 2). The clusters were significantly different from one another in their average prevalence of COVID-19 variant cases and variant-specific prevalence, vaccine uptake, and public health countermeasures (see Table 2). Multimedia Appendix 2 shows the frequency distribution of the variables after Benjamini-Hochberg correction for post hoc pairwise comparisons.

Yukon, Northwest Territories, and Nunavut—the first cluster—had a moderate prevalence of VOCs, high vaccine uptake (fully vaccinated), and low countermeasures. The 4 Atlantic provinces, New Brunswick, Newfoundland and Labrador, Nova Scotia, Prince Edward Island, and Quebec—the second cluster—had a low prevalence of VOCs, low vaccine uptake (fully vaccinated), moderate mobility, and moderate countermeasures. The 4 western provinces, British Columbia, Alberta, Saskatchewan, and Manitoba, along with Ontario—the third cluster—showed a high prevalence of VOCs, moderate vaccine uptake, high mobility, and high countermeasures.

**Table 2:** Characteristics of clusters from spatial hierarchical clustering analysis of mitigating factors.

Characteristics	All clusters (n=13)	Cluster 1: YT^a^, NT^b^, NU^c^ (n=3)	Cluster 2: NB^d^, NL^e^, NS^f^, PE^g^, QC^h^ (n=5)	Cluster 3: AB^i^, BC^j^, MB^k^, ON^l^, SK^m^ (n=5)	*P* value^n^
**Variants of concern (cases per 1 million population), median (IQR)**					
All (B.1.1.7, B.1.351, P.1, and B.1.617.2)	900.43 (236.55 to 4597.61)	532.9 (189.61 to 1705.96)	236.55 (175.20 to 374.68)	5461.33 (4597.61 to 9919.34)	.01
Only Alpha B.1.1.7	791.63 (230.16 to 4371.68)	532.9 (71.1 to 1683.8)	230.16 (162.68 to 359.31)	5200.06 (4371.68 to 9530.06)	.01
Only Beta B.1.351	11.53 (0 to 32.59)	0	11.53 (5.11 to 12.25)	32.59 (26.20 to 35.16)	.04
Only Gamma P.1	56.9 (1.28 to 195.96)	22.16 (0 to 118.51)	1.28 (1.02 to 1.92)	280.38 (195.96 to 609.52)	.01
Only Delta B.1.617.2	4.08 (0 to 56.84)	0	2.04 (1.92 to 4.08)	73.14 (56.84 to 80.47)	.007
2-dose vaccine coverage (%), median (IQR)	13.83 (10.85 to 18.89)	58.53 (40.39 to 61.56)	10.49 (5.67 to 10.85)	15.85 (13.83 to 17.98)	–^o^
Stringency index (%), mean (SD)	67.411 (11.41)	58.49 (9.78)	68.61 (10.09)	71.57 (12.55)	–^o^
Mobility index (z-score change), median (IQR)	0.41 (–1.04 to 1.25)	–2.09 (–3.22 to –1.04)	0.41 (–1.04 to 0.7)	1.25 (1.19 to 1.31)	–^o^

^a^YT: Yukon.

^b^NT: Northwest Territories.

^c^NU: Nunavut.

^d^NB: New Brunswick.

^e^NL: Newfoundland and Labrador.

^f^NS: Nova Scotia.

^g^PE: Prince Edward Island.

^h^QC: Quebec.

^i^AB: Alberta.

^j^BC: British Columbia.

^k^MB: Manitoba.

^l^ON: Ontario.

^m^SK: Saskatchewan.

^n^Intercluster differences assessed with the Kruskal-Wallis test.

^o^Analyses of the differences in vaccine coverage, stringency index, and mobility index across the clusters were not conducted because they contributed to the cluster analysis. Probabilistic assessment of the differences of these 3 variables across the clusters, therefore, was inappropriate.

#### Cluster Profile 1 (Yukon, Northwest Territories, and Nunavut; 23% of Variance): Moderate Prevalence of VOCs, High Vaccine, Low Stringency, and Low Mobility

Compared with the other clusters, the Northwest Territories, Nunavut, and Yukon were characterized by a moderate prevalence of aggregated VOCs and the Alpha variant (533 per 1 million population) and the Gamma variant (22 per 1 million population). It is important to note that the Beta and Delta variants had not been identified in the 3 territories of cluster 1. In addition, cluster 1 had the highest proportion of Canadians who had received 2 doses of COVID-19 vaccines (58.5%), lowest mobility index (–2.1), and lowest stringency index (58.5%).

#### Cluster Profile 2 (New Brunswick, Newfoundland and Labrador, Nova Scotia, Prince Edward Island, and Quebec; 38.46% of Variance): Low Prevalence of VOCs, Low Vaccination, Moderate Stringency, and Moderate Mobility

Compared with the other clusters, cluster 2 was characterized by the lowest prevalence of aggregated VOCs (237 per 1 million population), the Alpha variant (230 per 1 million population), and the Gamma variant (1 per 1 million population). However, it had relatively moderate levels of the Beta (12 per 1 million population) and Delta (2 per 1 million population) variants. In addition, cluster 2 had the lowest full vaccination coverage rates (10.5%), a moderate mobility index (0.4), and a moderate stringency index (68.6%).

#### Cluster Profile 3 (Alberta, British Columbia, Manitoba, Ontario, and Saskatchewan; 38.46% of Variance): High Prevalence of VOCs, Moderate Vaccination, High Stringency, and High Mobility

Compared with the other clusters, cluster 3 had the highest prevalence of VOCs (5461 per 1 million population) and variant-specific prevalences—Alpha (5200 per 1 million population), Beta (33 per 1 million population), Gamma (280 per 1 million population), and Delta (73 per 1 million population). Also, cluster 3 had moderate fully vaccinated coverage rates (15.9%), the highest mobility index (1.3), and the highest stringency index (71.6%).

### COVID-19 Variants of Concern and Vaccine Uptake

Figure 1 shows the distribution of VOCs and the proportion of Canadians who received the complete schedule of COVID-19 vaccine by province. Provinces shown in the darkest color (towards the top right in the legend in map) have relatively high VOC prevalences and high vaccination rates; those in the lightest color (bottom left) have low VOC prevalences and low vaccination rates.

Of all provinces and territories, Alberta was classified as having a marginally higher vaccine rate and high VOC prevalence: 18.9% of people fully vaccinated and VOC prevalence of 10,848 per 1 million population. Ontario (9919 per 1 million population), Saskatchewan (5461 per 1 million population), and Manitoba (4598 per 1 million population) had the high prevalences of VOCs and relatively moderate vaccine uptake rates (Ontario: 13.8%; Manitoba: 15.9%; and Saskatchewan: 18%).

At the opposite end, meaning low VOC prevalences and low vaccine rates, the Atlantic provinces with low VOC prevalences and low vaccine rates were Nova Scotia (VOC prevalence of 90 per 1 million population and 5.6% vaccine rate), Prince Edward Island (175 per 1 million population and 10.9%), New Brunswick (237 per 1 million population and 10.5%), and Newfoundland and Labrador (374 per 1 million population and 5.7%).

Québec and British Columbia had moderate VOC prevalences and vaccine uptake rates (Quebec, VOC prevalence of 900 per 1 million population and vaccine rate of 11.9%, and British Columbia, VOC prevalence of 3626 per 1 million population and vaccine rate of 12.8%). Yukon had a low prevalence of VOCs (190 per 1 million population) and high vaccine rate (41.6%), followed by the Northwest Territories (1706 per 1 million population and 58.53%) and Nunavut (533 per 1 million population and 40.4%).

### COVID-19 Variants of Concern and Public Health Countermeasures

Figure 2 shows the relationship between the prevalence of VOCs and government stringency measures to curb COVID-19. The provinces of Ontario and Manitoba showed higher prevalences of VOCs (9919 and 4598 per 1 million population, respectively) and high scores on the stringency index—Ontario: 90.7% and Manitoba: 75.9%. Saskatchewan recorded a higher prevalence of VOCs (5461 per 1 million population) and lower stringency (57.9%). Alberta had a high prevalence of VOCs (10,848 per 1 million population) and moderate stringency (68.5%).

The province of Quebec had a moderate level of VOC prevalence (900 per 1 million population) with high stringency (73.2%). British Columbia and Northwest Territories had moderate prevalences of VOCs (3626 per 1 million population and 1706 per 1 million population, respectively) and moderate levels of stringency—British Columbia: 64.8% and Northwest Territories: 64.8%.

At the low end of VOC prevalence, Yukon, Prince Edward Island, and New Brunswick had lower stringency—Yukon: 47.2%; Prince Edward Island: 58.3%; and New Brunswick: 58.3%. Nova Scotia reported a lower prevalence of VOCs (90 per 1 million population) and relatively higher stringency (81.5%), and Newfoundland and Labrador had a lower prevalence of VOCs, at 374 per 1 million population, and moderate stringency, at 71.8%.

### COVID-19 Variants of Concern and Mobility Index

Figure 3 presents the association between the prevalence of VOCs and changes in movement of people in relation to the beginning of the pandemic (mobility index). Among the provinces reporting higher VOC prevalences, populations in Ontario and Alberta showed higher mobility indices—Ontario: 3.0 and Alberta: 1.3. In this higher-prevalence VOC group, Manitoba and Saskatchewan populations had moderate mobility indices—1.2 and 0.2, respectively.

Among the provinces and territories reporting moderate VOC prevalences, Quebec and British Columbia recorded higher mobility indices—Quebec: 2.1 and British Columbia: 1.3—while Northwest Territories (–1.0) and Nunavut (–3.2) had lower mobility indices.

Two of the 4 Atlantic provinces, Prince Edward Island and Newfoundland and Labrador, and Yukon recorded low VOC prevalences and low mobility indices—Prince Edward Island at –2.7, Newfoundland and Labrador at –1.0, and Yukon at –2.1. Nova Scotia and New Brunswick recorded low prevalences of VOCs and moderate mobility, at 0.7 and 0.4, respectively.

## Discussion

### Principal Findings

This study shows markedly elevated prevalence of COVID-19 VOCs in Canada and wide geographical variation across the provinces and territories. We observed that the provinces in cluster 3—the Pacific region, Central Canada, and the Prairie provinces—were hot spots of VOCs as determined by the highest cluster-level prevalence of aggregated and variant-specific VOCs. We also observed that there was “north-south” disparity between the territories and the provinces in the prevalence of variant-specific VOCs, vaccine coverage, and public health countermeasures.

As with most COVID-19 research, this study sought to understand the disease as it is unfolding: specifically, COVID-19 VOCs in Canadian provinces and territories. By June 15, 2021, the daily new case rates in all Canadian provinces and territories were declining, indicating the resolution phase of the third wave of the epidemic curve. Many provinces set in motion plans to relax public health countermeasures, relying on vaccination rates as the criterion for reopening. At the same time, however, there was since-validated concern that the VOCs, in particular the Delta variant, could trigger widespread outbreaks, especially among the unvaccinated or partially vaccinated.

Across Canadian provinces and territories, we have shown a pattern of VOC spread, vaccine uptake (both doses), and policy countermeasures that can be profiled in 3 clusters. The first cluster, comprising all 3 Canadian territories, is characterized by moderate VOC prevalence, higher degree of vaccine uptake, and lesser degree of governmental countermeasures. The second cluster, comprising the Atlantic provinces of Nova Scotia, Prince Edward Island, Newfoundland and Labrador, and New Brunswick, along with the province of Quebec, is characterized by low VOC prevalence, low vaccine uptake, and moderate stringency of countermeasures. The third cluster, comprising Ontario, Manitoba, Saskatchewan, Alberta, and British Columbia, is characterized by high prevalence of VOCs, moderate vaccine uptake, and more stringent countermeasures. Strikingly, intercluster disparity in the prevalence of VOCs is more evident for cluster 3, making the provinces in that group VOC hot spots.

As governments across Canada continue to enact plans for easing public health countermeasures, there is concern that provinces and territories need to have much higher rates of vaccination with both primary and booster doses. Although per capita case rates are declining across Canada, there has been a recent increase in the highly transmissible VOCs, Delta and Omicron, in provinces [35,36]. These recent regional outbreaks were seen mostly among unvaccinated people [37].

As Canadian health officials strive to get as many people vaccinated within the shortest time possible, policy countermeasures may need to be further calibrated depending on the local spread of VOCs and vaccine hesitancy and refusals [38,39]. There have been several alarms raised that the B.A2 subtype of Omicron variant could become dominant if an insufficient proportion of the population did not complete the full COVID-19 primary vaccination regimen and receive booster doses [13]. In a past study conducted in Scotland, the first dose was shown not to confer complete immunity against the emerging variants versus the Alpha variant; however, full vaccination (2 doses) combined with booster shots improves immune effectiveness [38]. Our present study confirms that, in the postvaccine period in Canada (ie, December 2020), vaccination coverage rates are uneven across the country.

At least three strains of VOC with different transmission risks and responses to COVID-19 vaccines were concomitantly identified in more than half of the provinces. Like in the United States [15], in the first quarter of 2021, the dominant variant in Canada was the Alpha variant. According to Davies et al [14], the Alpha variant had a high reproductive number (43%-90%). However, a more worrying observation during the study period was the identification of 2 highly virulent variants (Beta and Delta) in 77% of the provinces. Due to the key mutations at E484K and K417N receptor-binding sites of the spike proteins for Beta [10-13] and L452R receptors for Delta [40], both variants are capable of escaping recognition by neutralizing antibodies (nAbs), thus evading both natural and vaccine-induced immunity. Also, T478K is not well recognized as responsible for immune evasion for Delta variants and may, like N501Y, be more relevant for angiotensin-converting enzyme 2 (ACE2) binding; however, additional mutations at K417N have been reported for the Delta variant [41]. Given the relatively high prevalence of the Gamma variant in some provinces or territories (ie, British Columbia, Alberta, Ontario, Saskatchewan, Manitoba, and Yukon), the Gamma variant also evades the immune system (but less so than the Beta variant) through a significant change in an nAb epitope (E484K and K417T).

This observation has far-reaching consequences on health outcomes and prolongation of the epidemic due to new infections. Considering the high prevalence of Beta, Gamma, Delta, and Omicron variants in some provinces (ie, Ontario, Quebec, Alberta, British Columbia, Saskatchewan, and Manitoba), stakeholders should continue to emphasize 2-dose vaccine uptake combined with booster shots and maintenance of public health countermeasures such as mask wearing and social distancing. Although the structural and operational barriers to vaccination (eg, vaccine stock shortages, long wait times, and vaccine refusal or hesitancy) need to be tackled, people must be adequately sensitized to complete the second and booster doses to offer full population protection against the VOCs (especially Beta, Gamma, Delta, and Omicron), thereby reducing future risk of new variants.

The discordance between VOC prevalence and stringency measures warrants cautious interpretation. Our analysis could not show whether the relaxation of countermeasures in the territories of Canada was informed by declining COVID-19 cases and progressive vaccine rollout, or vice versa. This inverse relationship, which is quite possible, means the timing of implementation of polices in relation to the changing epidemiological contexts of the pandemic is important. The degree of social compliance with government stringency measures might also depend on seasonality effects. Stringency measures may reduce COVID-19 incidence not only directly, for example, by reducing mobility, exposure, and public circulation but also indirectly through weather on mobility patterns [18,42]. Although we attempted to reduce the effect of weather by taking the average of mobility indices from January 2021 to June 2021, there might be some residual effects because of the varying daily temperatures across the provinces and territories. In our study, we observed low mobility index and stringency index in the territories, in contrast to the provinces.

At the time of conducting this study, no comprehensive time series data were easily available for Canada. Further investigation with time series will shed more light on the observed phenomenon. However, we could establish that successful immunization campaigns and reducing human mobility in the territories in northern Canada have played a role in lowering VOC cases. Fast tracking second-dose vaccination not only is key to return to normalcy but also has been shown in previous studies [14,43] to curtail transmission of COVID-19 variants.

Our results noted some spatial outliers (ie, discordant areas) for the relationship between the selected public health measures and VOCs in Ontario and in the Canadian Prairie provinces. To optimize public health impacts, these provinces need to revisit some of their approaches and reprioritize specific interventions. It is also noteworthy to further examine the intraprovincial variations between the public health measures and patterns of VOCs for the spatial outliers.

### Strengths and Limitations

The novelty of this study is that no known published study has described spatial clusters based on VOCs, vaccination coverage, and public health measures for Canadian jurisdictions. Overall, this study contributes to the current information needs to guide stakeholders on preventive measures to curtail VOCs during subsequent waves of the COVID-19 pandemic. Specifically, evidence from this study could serve as a comparison point for informing interventions for future variant-driven outbreaks and surges. Also, the bivariate choropleth map eased readability of spatial patterns, compared with proportional symbol maps. This study has some limitations. The recommendations for administration of different vaccine products (eg, messenger ribonucleic acid [mRNA]–based, viral vector–based) have been quite fluid in Canada; we have not taken into consideration the spatial associations between different vaccine products and VOCs. Further research is needed to specifically assess the geographical patterns of vaccine products and VOCs, especially for the Beta, Gamma, Delta and Omicron variants. As mentioned, this is an ongoing information need, rather than a one-time project.

The biggest limitation, however, is the time lag and the uneven testing, detecting, and sequencing efforts to identify VOCs in Canada. Whole genomic sequencing (WGS) efforts to identify VOCs by large volumes are currently lagging in Canada. Also, there is selection bias of the samples that are sent for sequencing. The provinces and territories have different criteria for sending samples for sequencing, which could delay detection and bias the proportion of VOCs associated with the Beta and Gamma variants. For example, in Ontario, the WGS was triggered from quantitative polymerase chain reaction (qPCR) testing for E48K. The samples with the E48K mutations were prioritized for WGS. However, in May 2021, the WGS algorithm changed to randomly selected sampling of 10% positives, with the proportion then increasing to 50% and now to all positives. This calls for new and rapid detection methods for VOCs even as Canada continues to fully vaccinate its populations. Also, there is possibility of systematic bias in the Google mobility index being underestimated among people without smartphones, who opted-out of the Google’s location history feature, or who had poor internet connection (especially in the territories). Due to how the mobility patterns were captured by Google LLC—geographical jurisdiction—it is challenging to delineate international travels and interprovincial and territorial mobility.

### Conclusions

This study found that COVID-19 VOCs in Canadian provinces and territories, to date, show discernible geographical clustering patterns: The territories recorded low VOC prevalences, Atlantic provinces and Quebec recorded moderate VOC prevalences, and the Western provinces and Ontario recorded high VOC prevalences. A fuller picture of VOC emerges when its prevalence is correlated with the proportions of populations having received 2 doses of vaccines, governmental countermeasures, and mobility. The implementation of COVID-19 public health measures including mass full vaccination of populations are key to controlling VOC prevalence rates in Canada. Surveillance of VOCs should continue across Canada, while accelerating the rollout of second and booster doses of vaccines. Achieving a balance in relation to lifting and relaxation of public health countermeasures and full-dose vaccine coverage is prudent to preempt any VOC-driven COVID-19 surges.

## Appendix

Multimedia Appendix 1 Description of variables.

Multimedia Appendix 2 Post-hoc pairwise comparison of the clusters.

## Abbreviations

ACE2: angiotensin-converting enzyme 2
CIHR: Canadian Institutes of Health Research
CoVaRR-Net: CIHR Coronavirus Variants Rapid Response Network
mRNA: messenger ribonucleic acid
nAbs: neutralizing antibodies
OxCGRT: Oxford COVID-19 Government Response Tracker
PCA: principal component analysis
qPCR: quantitative polymerase chain reaction
VOC: variant of concern
WGS: whole genomic sequencing

## Data Availability

Data on the cumulative number of variant of concern (VOC) cases can be downloaded from CTV News COVID-19 variants of concern tracker [7], and vaccine coverage rates are available at the COVID-19 Vaccination Tracker [21]. Provincial- and territorial-level population estimates are available from Statistics Canada [8]. The stringency index can be obtained at the Oxford COVID-19 Government Response Tracker (OxCGRT) website [23]. The mobility reports can be downloaded from Google LLC [24]. Researchers who require access to the study data can contact the corresponding author for further information.
