# Supplementary material for: COVID-19 Vaccination and Public Health Countermeasures on Variants of Concern in Canada: Evidence From a Spatial Hierarchical Cluster Analysis
Source: JMIR Public Health Surveill. 2022 May 31;8(5):e31968. doi: 10.2196/31968 (PMC9159466; doi:10.2196/31968)
Supplement: Multimedia Appendix 1 [file publichealth_v8i5e31968_app1.docx]

**Multimedia Appendix 1: Description of variables**

| Variable | Description | Source |
| --- | --- | --- |
| COVID-19 variants of concern (VOC) prevalence | Cumulative number of VOC cases  2021 Quarter 1 Territorial- and Provincial-level population estimates | CTV News COVID-19 variants of concern tracker [7]  Statistics Canada [8] |
| Vaccine coverage | Percentage of Canadian population fully vaccinated against COVID-19 | COVID-19 Vaccination Tracker [21] |
| Community stringency index  (9 indicators) | 1. School and workplace closures, 2. restrictions on public transport, 3. cancellation of public events and gatherings, 4. stay-at-home policies, 5. travel restrictions (interprovincial, intraprovincial and international), 6. public information campaigns, 7. testing policies, 8. contact tracing, and 9. face covering | Oxford COVID-19 Government Response Tracker (OxCGRT) website [23] |
| Community mobility index  (6 indicators) | Mobility trends for:   1. **Retail** (restaurants, cafes, shopping centers, theme parks, museums, libraries, and movie theaters) 2. **Groceries** (grocery markets, food warehouses, farmers markets, specialty food shops, drug stores, and pharmacies) 3. **Parks** (local parks, national parks, public beaches, marinas, dog parks, plazas, and public gardens 4. **Transit** (public transport hubs such as subway, bus, and train stations) 5. **Workplace** 6. **Residential areas** | Google LLC [24] |
