# Supplementary material for: COVID-19 Vaccination and Public Health Countermeasures on Variants of Concern in Canada: Evidence From a Spatial Hierarchical Cluster Analysis
Source: JMIR Public Health Surveill. 2022 May 31;8(5):e31968. doi: 10.2196/31968 (PMC9159466; doi:10.2196/31968)
Supplement: Multimedia Appendix 2 [file publichealth_v8i5e31968_app2.docx]

**Multimedia Appendix 2: Post-hoc pairwise comparison of the clusters**

| Variant | Comparison | BH-test | p-value** |
| --- | --- | --- | --- |
| All (B.1.1.7, B.1351, P.1 and B.1.617) | Cluster 1 vs. cluster 2  Cluster 1 vs. cluster 3  Cluster 2 vs. cluster 3 | 0.66  -1.88  -2.92 | 0.256  **0.046**  **0.005** |
| Only Alpha B.1.1.7 | Cluster 1 vs. cluster 2  Cluster 1 vs. cluster 3  Cluster 2 vs. cluster 3 | 0.28  -2.11  -2.76 | 0.389  **0.026**  **0.009** |
| Only Beta B.1351 | Cluster 1 vs. cluster 2  Cluster 1 vs. cluster 3  Cluster 2 vs. cluster 3 | -1.57  -2.6  -1.19 | 0.088  **0.014**  0.116 |
| Only Gamma P.1 | Cluster 1 vs. cluster 2  Cluster 1 vs. cluster 3  Cluster 2 vs. cluster 3 | 0.38  -2.05  -2.81 | 0.354  **0.03**  **0.008** |
| Only Delta B.1.617.2 | Cluster 1 vs. cluster 2  Cluster 1 vs. cluster 3  Cluster 2 vs. cluster 3 | -1.14  -3.03  -2.18 | 0.127  **0.004**  **0.022** |

***Benjamini-Hochberg adjustment*

*Cluster-profile 1: Yukon, Northwest Territories, and Nunavut; cluster-profile 2: New Brunswick, Newfoundland and Labrador, Nova Scotia, Prince Edward Island and Quebec; cluster-profile 3: Alberta, British Columbia, Manitoba, Ontario and Saskatchewan*

After Benjamini-Hochberg correction for post-hoc pairwise comparison, clusters 1 and 3 significantly differed in terms of the overall prevalence of VOC (difference (Δ)= 4928 per 1 million population, p-value=0.046), Alpha (Δ=4667 per 1 million population, p-value=0.026), Beta (Δ=32 per 1 million population, p-value=0.014), Gamma (Δ=258 per 1 million population, p-value=0.03) and Delta B.1617 (Δ= 73 per 1 million population, p-value=0.004). Except for Beta variant, clusters 2 and 3 significantly differed for the overall prevalence (Δ=5225 per 1 million population, p-value=0.005), Alpha (Δ=4970 per 1 million population, p-value=0.009), Gamma (Δ=279 per 1 million population, p-value=0.008) and Delta (Δ=71 per 1 million population, p-value=0.02).
